# The Clock Input to the First Optic Neuropil of *Drosophila melanogaster* Expressing Neuronal Circadian Plasticity

**DOI:** 10.1371/journal.pone.0021258

**Published:** 2011-06-27

**Authors:** Milena Damulewicz, Elzbieta Pyza

**Affiliations:** Department of Cell Biology and Imaging, Institute of Zoology, Jagiellonian University, Krakow, Poland; Alexander Flemming Biomedical Sciences Research Center, Greece

## Abstract

In the first optic neuropil (lamina) of the fly's visual system, two interneurons, L1 and L2 monopolar cells, and epithelial glial cells show circadian rhythms in morphological plasticity. These rhythms depend on clock gene *period* (*per*) and *cryptochrome* (*cry*) expression. In the present study, we found that rhythms in the lamina of *Drosophila melanogaster* may be regulated by circadian clock neurons in the brain since the lamina is invaded by one neurite extending from ventral lateral neurons; the so-called pacemaker neurons. These neurons and the projection to the lamina were visualized by green fluorescent protein (GFP). GFP reporter gene expression was driven by the *cry* promotor in *cry*-GAL4/UAS-GFP transgenic lines. We observed that the neuron projecting to the lamina forms arborizations of varicose fibers in the distal lamina. These varicose fibers do not form synaptic contacts with the lamina cells and are immunoreactive to the antisera raised against a specific region of *Schistocerca gregaria* ion transport peptide (ITP). ITP released in a paracrine way in the lamina cortex, may regulate the swelling and shrinking rhythms of the lamina monopolar cells and the glia by controlling the transport of ions and fluids across cell membranes at particular times of the day.

## Introduction

In the visual system of flies several processes show circadian oscillations. The rhythms have been detected in the retina and in the first optic neuropil (the lamina). The retina possesses its own circadian oscillators in the photoreceptor cells, while in the lamina the glial cells are possible circadian oscillators [1]–[3]. In the lamina, circadian rhythms have been detected in changes of the number of several structures in the photoreceptor terminals [4] and of synaptic contacts [5], and in morphological plasticity of interneurons [6]–[8] and glial cells [9]. In three fly species, *Musca domestica*, *Calliphora vicina* and *Drosophila melanogaster*, axons of the first order interneurons, L1 and L2 monopolar cells change their girth during the day [6], [7], [10], [11]. Moreover, L2 dendritic trees examined in *D. melanogaster*, show circadian oscillation in their shape and size and are largest at the beginning of the day [8]. The function and mechanisms of circadian plasticity of monopolar cell axons and dendrites in the fly's visual system are only partly known. In the housefly, it has been found that injections of various lamina neurotransmitters mimic the morphological changes which were observed in the L1 and L2 axons [12]. We have also detected that protein synthesis is involved in cell swelling. Whereas, disruption of microtubules and actin microfilaments during the night, blocks shrinkage of the L1 and L2 axons and decreases the tetrad synapse number formed between the photoreceptor terminals and the lamina cells [13]–[15]. Changes in the L1 and L2 axon size are correlated with the pattern of the locomotor activity of the fly species and with the number of tetrad synapses. In *D. melanogaster*, the locomotor activity pattern is bimodal. There are two peaks of activity; in the morning and in the evening. A similar pattern of changes was observed in the cross-sectional area of the L1 and L2 axons which was larger at the beginning of the day and at the beginning of the night [7]. Using *D. melanogaster* arrhythmic null mutant of *period* (*per)* gene, *per* ^01^, we have found that this mutation abolishes the circadian rhythm in morphological changes of L2 dendritic trees. In turn, mutation of *cryptochrome* (*cry*) gene, *cry* ^b^, encoding the circadian photoreceptor protein CRYPTOCHROME (CRY), changes the pattern of the rhythm [8]. On the other hand, severing the housefly's optic lobe from the rest of the brain also abolishes the rhythmic swelling and shrinking of L1 and L2. This result indicates that clock neurons located in the brain, are involved in the generation of circadian rhythms in the morphological changes of monopolar and glial cells in the lamina [16], [17].

In the brain of *D. melanogaster*, there are about 150 clock neurons grouped into 7 sets: 3 dorsal and 4 lateral, on each side of the brain [18], [19]. The dorsal neurons (DNs) are divided into 3 subgroups: 17 DN_1_s, 2 DN_2_s and 40 DN_3_s. The lateral neurons form 4 groups: 6 dorsal lateral neurons (LN_d_s), 5 small ventral lateral neurons (s-LN_v_s), 4–5 large ventral lateral neurons (l-LN_v_s) and lateral posterior neurons (LPNs). The s-LN_v_s maintain circadian rhythm in locomotor activity, in constant darkness (DD). In day/night (LD 12∶12) conditions, the s-LN_v_s control the morning peak of activity. The LN_d_s and 5^th^ s-LN_v_ associate with DNs to support the evening peak of activity [19], [20]–[22]. Less is known about the function of DNs. The DN_1_s are probably involved in integration of light and temperature inputs controlling behavioral rhythms [23], [24]. The last group of pacemaker neurons, the lateral posterior neurons (LPNs) seem to be important for synchronization to the temperature cycle [18], [25], [26].

The small LN_v_s, with the exception of the 5^th^ s-LN_v_, produce pigment-dispersing factor (PDF), a circadian neurotransmitter [27], [28], which is important for synchronization in the phase and amplitude molecular oscillations of clock neurons within the circadian system [29], [30]. PDF in the s-LN_v_s is probably non-amidated and is transported and released in the dorsal protocerebrum in a rhythmic manner [31]. It has been suggested that large LN_v_s produce C-terminally amidated PDF, and that this type of PDF has a longer half-life and is more active than the non-amidated form [32]. This neuropeptide is released in the medulla of *D. melanogaster* [33] but its receptors have also been detected at the base of the eye [34]. PDF may also synchronize peripheral clocks and transmit circadian information to non-clock cells [12], [34]–[36]. The 5^th^ s-LN_v_ does not express PDF but it does express the ion transport peptide (ITP) [37]. Among the LN_v_s this is the only neuron that plays a role in regulating the evening activity peak [22].

Light is the most important donor of time perceived by several types of photoreceptors in *D. melanogaster*. These photoreceptor types include the retinal photoreceptors of the compound eyes, the ocelli on the top of head, the Hofbauer-Buchner eyelet in the lamina and the cellular photoreceptor – CRY. The last photoreceptor seems to be the most important for light entrainment. This is because strong phase shifts of the rhythms are caused by blue light for which CRY has the maximal absorption [38]. CRY resets the clock every morning, after photon absorption and binding TIMELESS (TIM) protein encoded by another clock gene *tim* [39]. Then, TIM is ubiquitinated and degraded in proteasomes [40]. This process also leads to degradation of the PER that forms heterodimers with TIM [41]. In this way, the molecular clock in the pacemaker cells is reset by light. CRY may also function in the molecular mechanism of the circadian clock in peripheral oscillators. CRY might function as the circadian repressor of two clock transcription factors; CLOCK (CLK) and CYCLE (CYC), which form heterodimers and regulate *per* and *tim* transcription [42]–[44].

In our earlier study we observed that PER and CRY are needed to maintain the circadian rhythms in the lamina of *D. melanogaster* [8]. However, the circadian input to the lamina was unknown. The large LN_v_s form a dense network of PDF-immunoreactive processes, in the medulla of the optic lobe, but this network terminates in the margin of the medulla. In the present study, we show for the first time, that this input exists and that it originates from the LNs. This input uses an ITP-like peptide as a neurotransmitter, an unknown yet signaling pathway in the circadian system.

## Results

**Figure 1:**
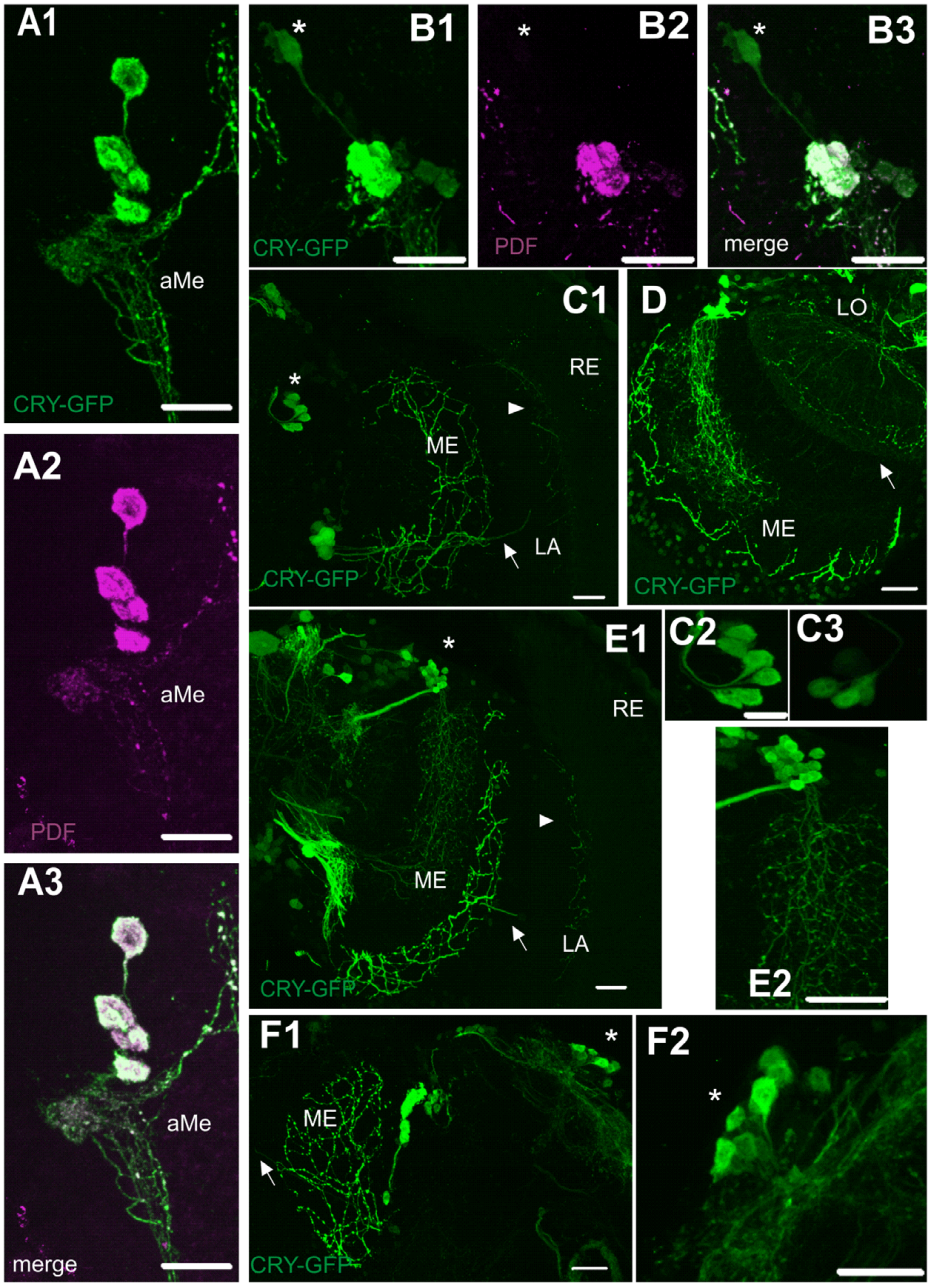
Localization of CRY-positive cells in the brain of *Drosophila melanogaster*. Flies were examined at four ZTs: ZT1, ZT4, ZT13 and ZT16 (ZT0 – the beginning of the day and ZT12 – the beginning of the night) but images shown in the Fig. 1 were obtained from the brain of individuals collected for experiments at different ZTs. ZT for each image is given in brackets. A1–3: Four large ventral lateral neurons (l-LN_v_s), four small LN_v_s (s-LN_v_s) and an arborization in the accessory medulla (aMe) immunoreactive to PDF (magenta) and labeled with *cry*-GAL4-driven GFP (green) (ZT1). B1–3: Double-labeling of the LN_v_s with PDF antiserum (magenta) and *cry*-GAL4-driven GFP (green) (ZT16). The 5^th^ s-LN_v_ expresses GFP but not PDF (asterisk). C1: *cry*-GAL4-driven GFP expression in the optic lobe: LN_v_s and LN_d_s (asterisk). The LN_v_s send processes to the medulla (ME) and a single projection to the lamina (LA) which divides and terminates in the lamina cortex (arrows) (ZT4). C2–3: CRY-positive dorsal lateral neurons (LN_d_s). Out of 6 LN_d_s (C2) (ZT4), 3–4 cells have a higher (by 45–80%) intensity of GFP than other LN_d_s in most preparations (C3) (ZT13). D: CRY-positive network of processes in the lobula (LO) (arrow). Processes from DN_3_s and LN_v_s in the medulla, also shown. E1–2: Dorsal neurons DN_3_s and LN_v_s. The LN_v_s project to the medulla (ME) and to the lamina (arrows) (ZT4). DN_3_s form a cluster of cells (asterisk) and send processes to the medulla (E2). F1–2: Network of processes of LN_v_s with varicosities in the medulla and in the lamina (arrow); CRY-positive DN_1_s (asterisk, F2) (ZT1). Scale bars: 20 µm; in C2: 10 µm.

Detected CRY-positive cells, using *cry*-GAL4 driven expression of GFP reporter gene, were found in the central brain and in the optic lobe. Labeling with anti-GFP, to strengthen GFP signal, showed a strong fluorescence in the ventral lateral neurons (LN_v_s) (Fig. 1A1–3, B1–3), in the dorsal lateral neurons (LN_d_s) (Fig. 1C1,2), and in the dorsal neurons DN_1_ and DN_3_ (Fig. 1D, E1,2, F1,2). GFP was not detected in the dorsal neurons DN_2_ and in the lateral posterior neurons (LPN).

**Figure 2:**
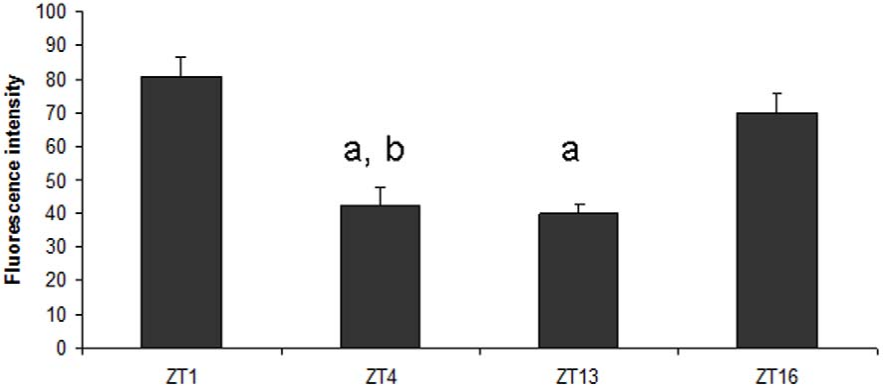
*Cry*-GAL4-driven GFP intensity measured at different time points (ZTs) in the 5^th^ s-LN_v_ cell body. Means ± SEM, a and b mean statistically significant differences between ZT1 and ZT16, respectively, and other time points. Statistics: Non-parametric ANOVA Kruskal-Wallis Range Test [N = 21; H = 11.755; p = 0.083].

Co-localization analysis of GFP and PDF-immunolabeling, showed that CRY is present in all LN_v_s; 4 large and 4 small PDF-positive LN_v_s, and in the 5^th^ s-LN_v_ PDF-negative (Fig. 1A1–3, B1–3). The small PDF-positive LN_v_s form a cluster of cells located next to each other in the accessory medulla (aMe). The 5^th^ s-LN_v_, however, is detached from this cell cluster and localized more dorsally in the brain. The large LN_v_s are located above the s-LN_v_s in the brain. The PDF-immunoreactive varicose processes of the large LN_v_s invade the medulla and these processes were also positive to *cry*-GAL4 driven GFP (Fig. 1C1). The intensity of GFP fluorescence was stronger in l-LN_v_s than in s-LN_v_s. The more intense fluorescence in the l-LN_v_s suggests a higher level of CRY expression in the l-LN_v_s. The intensity of GFP fluorescence in the LN_v_s was measured at the following four time points in the LD 12∶12 condition: ZT1, ZT4, ZT13 and ZT16, and the obtained results confirmed findings which have already been reported [45]. In l-LN_v_s, LN_d_s and in the 5^th^ s-LN_v_, the GFP level was higher than in other s-LN_v_s and in DNs. Moreover, the level of GFP in the 5^th^ s-LN_v_ was the highest at ZT1 and higher at ZT16 than in ZT4 and ZT13 (Fig. 2). The pattern of changes of *cry*-GAL4 driven GFP intensity in the 5^th^ s-LN_v_ resembles the pattern of the daily morphological changes of L2 dendritic trees [8]. Both rhythms show maximum at the beginning of the day. These observations of the CRY level changes in DNs and LNs were confirmed by using anti-CRY serum (data not shown).

GFP driven by *cry*-GAL4 also visualized all 6 LN_d_s as CRY-positive cells (Fig. 1C1,2). These cells form a specific cluster in which neurons are located next to each other in one “bunch” and each single cell has a connection with the anterior optic tract (AOT). Out of 6 LN_d_s, 3 cells had a higher intensity of GFP fluorescence (Fig. 1C3) (ZT1: 153.7±49 SEM, ZT4: 124.7±14 SEM, ZT13: 177.6±21 SEM, ZT16: 101.7±2 SEM). The other 3 cells showed a fluorescence intensity which was 53%, 20%, 55%, 38% lower at ZT1 (72.7±17 SEM), ZT4 (100.4±13 SEM), ZT13 (79.5±14 SEM), ZT16 (62.9±5 SEM), respectively. These differences were observed in 6–9 flies per time point.

**Figure 3:**
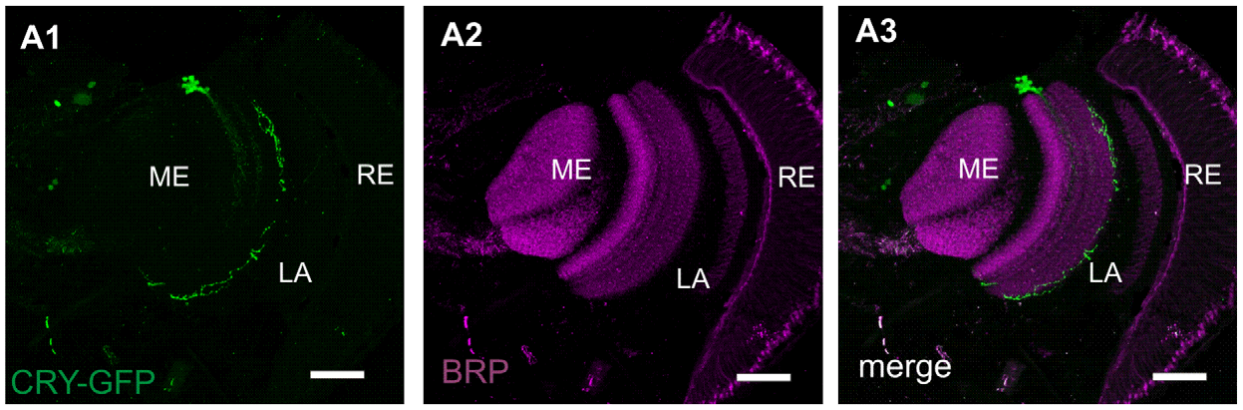
Dorsal neuron (DN_3_) projections to the medulla labeled with *cry*-GAL4-driven GFP (A1, green) and immunolabeled with BRP (A2, magenta) antiserum. Flies were examined at four ZTs: ZT1, ZT4, ZT13 and ZT16 (ZT0 – the beginning of the day and ZT12 – the beginning of the night) but images shown in Fig. 3 were obtained from the brain of individuals collected at ZT13. Projections from the DN_3_ to the medulla may form synaptic contacts since co-localization of BRP and these CRY-positive processes have been observed (A3). RE – retina, LA – lamina, ME – medulla. Scale bars: 20 µm.

The DN_3_s form a cluster in the dorsal brain, next to the LN_d_s. We found that DN_3_ of the dorsal neurons were all labeled with GFP, and that DN_3_ processes invaded the medulla (Fig. 1E1, 2). In the medulla, the DN_3_ projections form a dense network of processes between PDF-positive processes originating from the l-LN_v_s (Fig. 3A1–3). This DN_3_ network seemed to originate from a single thick process extending from the DN_3_ cluster of cells. The DN_3_ processes form synaptic contacts with the medulla neurons (Fig. 3A3).

A CRY-positive network of processes is also present in the lobula, the third optic neuropil (Fig. 1D). This network is clearly separated from the medulla network. There were no observed connections between the CRY-positive networks in the neuropils of both the lobula and medulla. The location of the cell bodies of the lobula processes is unknown.

**Figure 4:**
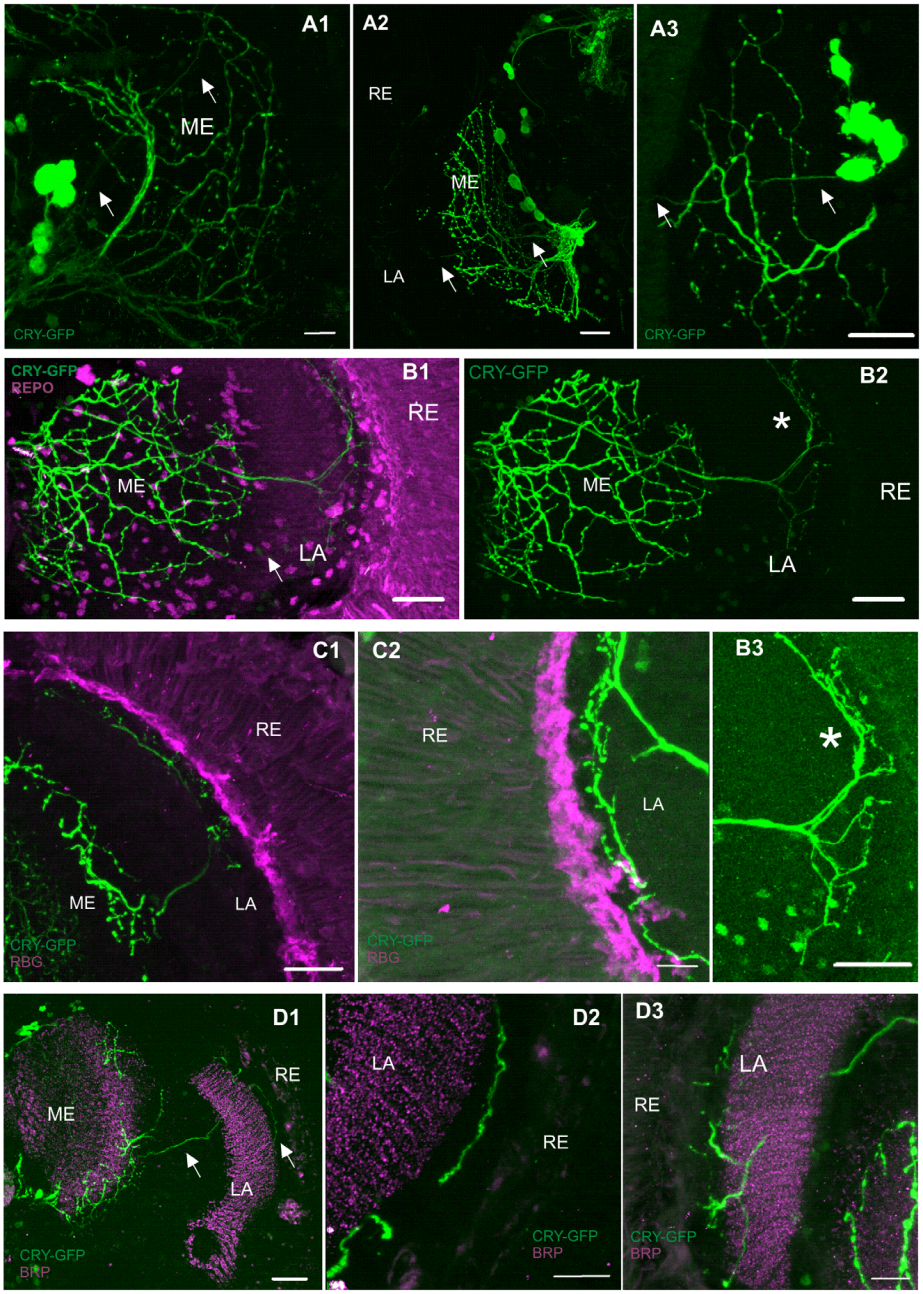
CRY-positive processes in the medulla and lamina of *Drosophila melanogaster*. Flies were examined at four ZTs: ZT1, ZT4, ZT13 and ZT16 (ZT0 – the beginning of the day and ZT12 – the beginning of the night) but images shown in Fig. 4 were obtained from the brain of individuals collected for experiments at different ZTs. ZT for each image is given in brackets. A1–3: *cry*-GAL4-driven GFP in the medulla and lamina. CRY-positive projection from lateral neurons (LN_v_s) passes the medulla, invades the lamina (arrows), and terminates in the lamina corte (A1: ZT16, A2: ZT13, A3: ZT1). B1–3: Cells labeled with *cry*-GAL4-driven GFP (green) and REPO-immunolabeled glial cells (magenta). Numerous cells in the medulla and lamina are CRY-positive, but REPO-negative (arrow). CRY-positive projection from the LN_v_s divides in the lamina cortex. The projection forms arborized processes with varicosities (asterisk) (ZT4). C1–2: Immunolabeling of the fenestrated glia with the antibody against DVMAT (magenta) and *cry*-GAL4-driven GFP in processes (green) in the lamina cortex. CRY-positive terminals do not invade the layer of the fenestrated glia (C2) (C1: ZT16, C2: ZT13). D1–3: Immunolabeling with BRP (magenta) antiserum and cells labeled with *cry*-GAL4-driven GFP (green). A neuron projecting from the LN_v_s to the lamina does not form synaptic contacts with the lamina cells (D2) (D1,2: ZT1, D3: ZT4). RE – retina, LA – lamina, ME – medulla. Scale bars: 20 µm.

We also detected CRY-positive processes in the lamina. These processes extend from the pacemaker neurons in the proximal medulla (Fig. 4A1–3). A single, straight neurite passes the medulla neuropil and invades the lamina, forming arborization of thin fibers in the lamina cortex (Fig. 4B3). These terminals are located near the retina, in the region of the somata of the lamina monopolar cells (Fig. 4B1,2). They do not extend to the retina, and terminate at the border of the fenestrated glia (Fig. 4C1,2). These CRY-positive processes in the lamina do not show any morphological changes during the day and during the night.

To examine possible synaptic contacts between CRY-positive terminals and the lamina cells, we used nc82 antibody against the active zone presynaptic protein Bruchpilot (BRP), to visualized presynaptic sites. Analysis of the co-localization of BRP and GFP at four time points, showed that CRY-positive terminals in the lamina do not form synaptic contacts with the lamina cells (Fig. 4D1–D3).

**Figure 5:**
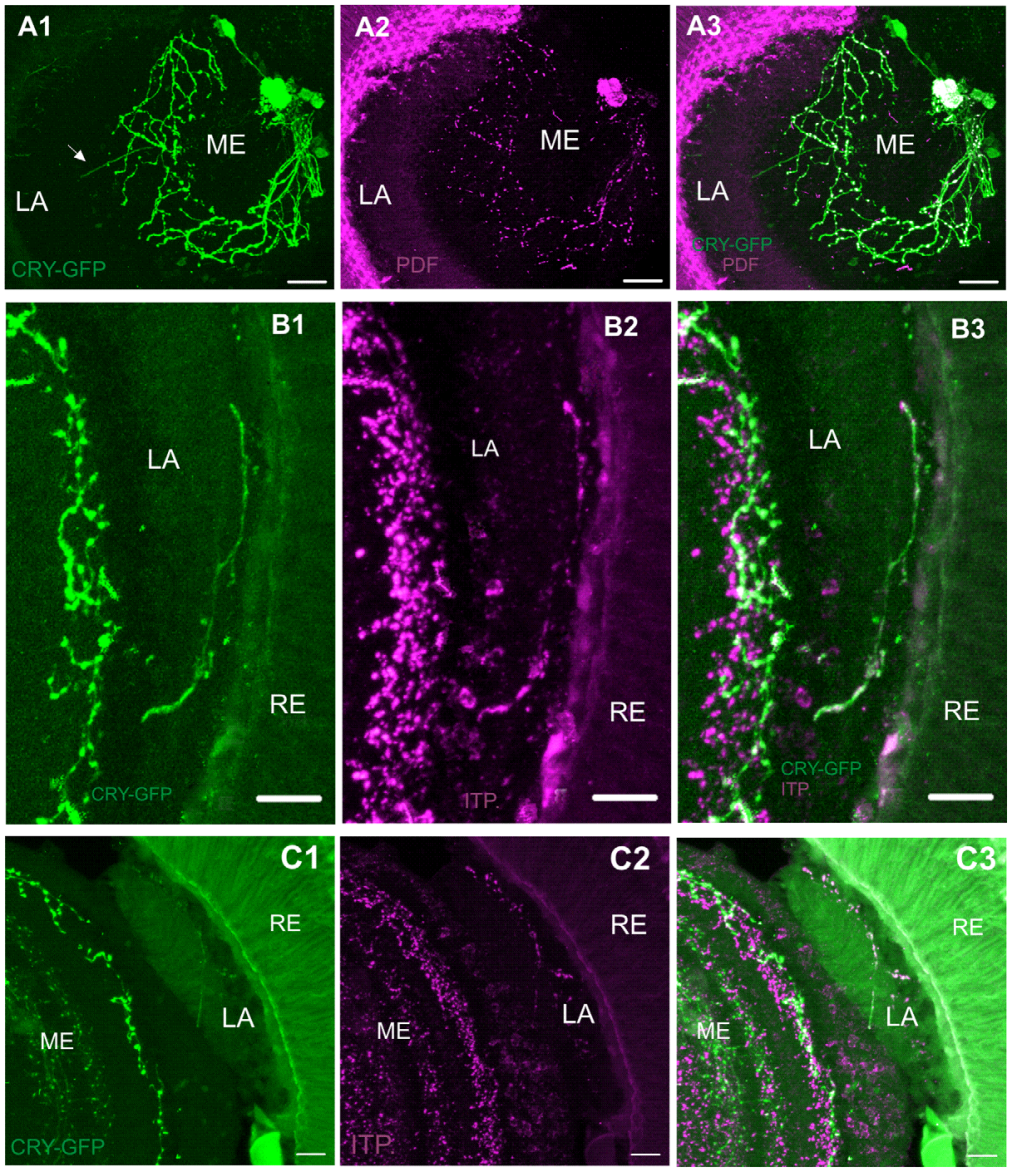
Localization of PDF and ITP neuropeptides in the optic lobe of *Drosophila melanogaster*. Flies were examined at four ZTs: ZT1, ZT4, ZT13 and ZT16 (ZT0 – the beginning of the day and ZT12 – the beginning of the night) but images shown in Fig. 5 were obtained from the brain of individuals collected for experiments at different ZTs. ZT for each image is given in brackets. A1–3: PDF- immunoreactive (magenta) and labeled with *cry*-GAL4-driven GFP (green) neurons. The 5^th^ s-LN_v_ is PDF-negative and PDF is also not present in CRY-positive projection invading the lamina (arrow) (ZT16). B1–3 (ZT1), C1–3: ITP-immunoreactive (magenta) and labeled with *cry*-GAL4-driven GFP (green) neurons. ITP- immunoreactivity co-localizes with CRY-positive processes in the lamina. The magenta staining in the retina is non-specific while in the medulla, ITP- immunoreactivity is both specific and unspecific (ZT13). RE – retina, LA – lamina, ME – medulla. Scale bars: 20 µm.

To determine the origin of the projection from the proximal medulla to the lamina, we used 100 µm vibratom sections and 3D reconstructions of neurons in the optic lobe. This method showed that the projection extends from the aMe, where the s-LN_v_s and l-LN_v_s somata are located (Fig. 4A1–3). Double labeling with anti-PDF serum showed the lack of PDF-immunoreactivity in this neurite in the medulla and in its terminals in the lamina (Fig. 5A1–3). The results suggest that the projection to the lamina does not originate from the l-LN_v_s or the four s-LN_v_s, but from the 5^th^ s-LN_v_. To verify if the projection originates from the 5^th^ s-LN_v_, we used antibody raised against a specific region of *Schistocerca gregaria* neuropeptide ion transport peptide (ITP) (residues 60–67; DEEEKFNQ) (a kind gift from Dr. Neil Audsley). In addition, we tested the antisera specific for ITP-L, made to residues 65–79 (IQSWIKQIHGAEPGV) of *S. gregaria* ITP (a kind gift from Dr. Neil Audsley) and to RLRWamide (short neuropeptide F – sNPF-3 and -4) (a kind gift from Dr. Jan A. Veenstra). The results showed the co-localization of CRY and Schgr-ITP only (Fig. 5B1–3, C1–3). To confirm the presence of ITP in the lamina we carried out ITP immunolabeling using wild-type flies (Canton-S). ITP-positive varicose fibers in the lamina cortex were detected.

In addition to observing GFP fluorescence in clock cells, we observed GFP fluorescence in many other neurons in the brain. Using the antibody against REPO protein – a specific marker of glial cells, we did not observe co-localization of GFP and REPO. This lack of co-localization indicates that CRY is probably not present in glial cells. Many CRY-positive cells, though, were located in the close vicinity of glial cells (Fig. 4B1–3). Finally we used the serum against the N- terminus of DVMAT [45] (a kind gift from Dr. Bernhard T. Hovemann), to label the fenestrated glia. We did this to find target cells for CRY-positive terminals in the lamina. These fibers, however, do not invade the fenestrated glia and terminate in the region of the L1 and L2 interneuron cell bodies (the lamina cortex) (Fig. 4C1,2).

## Discussion

In the present study we showed, for the first time, a single projection from the pacemaker cells in the brain to the lamina, in which several structural circadian rhythms have been detected [46]. Moreover, we found that this input probably originates from the 5^th^ small LN_v_. Since the 5^th^ s-LN_v_ does not express PDF, this cell is different from the other LN_v_s. The possibility that this process originates from other clock cells, for example from the LN_d_s, and extends to the aMe first, and next to the lamina cannot be excluded. A CRY-positive LN_d_, which is immunoreactive to ITP, could invade the lamina by passing the aMe first. This neuron, however, is also immunoreactive to sNPF, but the projection detected in the lamina is immunoreactive to ITP only. It indicates that this projection originates from the 5^th^ s-LN_v_, which is immunoreactive to ITP but not to sNPF. In our study, we examined GFP expression driven by *cry*-GAL4 in thin, 20 µm cryostat sections and thick 100 µm vibratom sections of the *D. melanogaster* brain. In most earlier studies on clock neurons and their projections, whole-mount preparations of the *Drosophila* brain were used, or the lamina was cut-off during preparation. Such procedures from previous studies meant that the very fine projection from the brain to the lamina could not be observed. We detected the projection by using 20 µm sections and collecting confocal optical sections at a 1 µm interval.

In several previous studies, it has been suggested that CRY is present in different types of clock neurons. These results have been obtained using various methods; *cry*-GAL4 driven GFP expression [18], [19], [47]–[50], *cry* mRNA *in situ* hybridization [48], immunolocalization [48], [50] and *cry* deletion mutants [50]. Using *cry*-GAL4 line and 20 µm sections of the *D. melanogaster* brain, we found that CRY is located in all s-LN_v_s, l-LN_v_s, LN_d_s, DN_1_s and DN_3_s but is absent in DN_2_s and LPNs. These results only partly confirm the results of earlier studies by Klarsfeld et al. [49], Helfrich-Förster et al. [19], Yoshii et al. [45] and Benito et al. [50]. Yoshii et al. [45] showed that LN_v_s but only some DN_1_, and three or four from the six LN_d_ are CRY – positive, while DN_2_, DN_3_ and LPNs are CRY-negative. Benito et al. [50] also did not detect CRY in DN_2_s and DN_3_s, and in about half of the LN_d_s and DN_1_, but *cry* promoter dependent reporter genes and *cry* mRNA can be detected in these neurons. In our study, all of LN_d_s showed GFP fluorescence in the *cry*-GAL4 strain, but only 3–4 cells were found to be CRY-immunopositive using antibodies [45]. In turn, using the *in situ* hybridization method, *cry* mRNA was not detected in those cells [48]. Since the pattern of *cry*-GAL4 driven GFP expression depends on the transgene insertion site and whether the first intron of the transgene has been inserted, Zheng et al. [51] examined spatial and circadian regulation of *cry*. They used a series of *cry*-GAL4 transgenes containing different portions of *cry* upstream and intron 1 sequences. This study showed that the first intron drives expression in eyes and antennae and that upstream sequences induce *cry* expression in brain clock neurons and in peripheral oscillators; in eyes and antennae. In addition, upstream sequences also induce expression of *cry*, in other non-clock cells in the optic lobe.

The results obtained using various methods suggest that in the case of CRY, translation and *cry* transcription may be specifically regulated. CRY-positive labeling in the 4^th^ LN_d_ was observed in flies kept for 5 days in constant darkness. Flies kept longer in this condition brought on weak staining in one of the DN_2_ neurons [45]. Thus, the level of CRY in this neuron may be very low, and the CRY level may only be detected after it has accumulated for several days in DD. It is possible, that in some of the LN_d_s, DN_1_ and DN_3_ *cry* expression is very low and protein is undetectable by the immunohistochemistry method, or that *cry* mRNA is unstable and CRY protein is not synthesized. Among six LN_d_s, three neurons, that show a strong signal of GFP in the brain cryostat sections used in our study, may correspond to CRY-positive cells detected in the studies of other authors. In turn, three LN_d_s with weak GFP in our preparations may correspond to CRY immunonegative cells [50]. These cells had about a 50% lower GFP level than the rest of the LN_d_s at all time points, except at ZT4 when their GFP fluorescence was lower by 20%.

Beside neurons, clock genes have also been detected in glial cells [45]. A subpopulation of glial cells in the brain of *D. melanogaster* have rhythmic expression of *per* gene, and they are necessary for maintaining circadian locomotor activity [52]. However, the presence of CRY in glia was not detected in our study. In the optic lobes, GFP driven by *cry*-GAL4 was observed in many non-clock cells in which the localization pattern was very similar to the distribution of glial cells. But these non-clock cells were not labeled with the antibody against REPO protein, a specific marker for glial cells. The REPO protein is required for glia development and differentiation [53] and has been detected in all types of glia in the adult brain of *D. melanogaster* [54]. The analysis of *cry*-GAL4 driven GFP and REPO immunolabeling showed no co-localization between CRY and REPO. However, in the close vicinity of GFP-positive cells, REPO-positive glial cells were observed. We obtained a similar result using the antibody against the *D. melanogaster* vesicular monoamine transporter (DVMAT), which enabled us to label the fenestrated glia in the optic lobe. These results suggest that CRY is present in non-clock neurons in the optic lobe, but not in glial cells.

In addition to localization of *cry*-GAL4 driven GFP in cell bodies of neurons, we also detected GFP processes invading three neuropils in the optic lobe. In the medulla, a dense network of processes originate from DN_3_s and their terminals seem to form synaptic contacts with not-yet identified target cells. The regular network of processes was also detected in the lobula but their origin is unknown. The most interesting finding is the projection of CRY-positive processes to the lamina. Although the lamina showed robust circadian remodeling of neuron morphology, a circadian input had not been previously detected. In the lamina, *per* is probably expressed in the epithelial glial cells, however, maintaining the lamina structural rhythms also requires *per* expression in the retina photoreceptors and in the LNs [55].

Beside PER, CRY is also important for circadian rhythms in the lamina. In our earlier study, we have shown that the circadian rhythm in morphological plasticity of L2 dendritic trees, is not present in *per* ^01^ mutant while its phase depends on CRY. In *cry* ^b^ mutant, the pattern of daily changes in size of the L2 dendritic tree was different than in wild-type Canton-S flies [8]. In males and females of Canton-S wild-type flies, the largest L2 dendritic tree was found at the beginning of the day. This daily pattern of the structural changes of L2 dendrite resembles the pattern of *cry* mRNA cycling in *D. melanogaster* heads and bodies [38], and in the 5^th^ s-LN_v_ detected in our study. Although the L2 dendritic tree is the largest at the beginning of the day in the distal lamina, its axon, as well as the axon of L1 monopolar cell, swell at the beginning of both day and night [7]. These changes have been detected in the proximal lamina. Moreover, the α–subunit of the Na^+^/K^+^-ATPase and subunits of the V-ATPase also show diurnal changes in abundance in the lamina. Such an occurrence indicates that circadian rhythms in cell structural plasticity are correlated with rhythmic changes in the level of proteins involved in the transport of ions [55], [56]. The rhythm in the α–subunit of the Na^+^/K^+^-ATPase level is bimodal with two peaks; in the morning and in the evening. This pattern is changed in the *cry* ^0^ mutant (Damulewicz M. and Pyza E., unpublished results). It indicates that CRY is not only important for the maintenance of the daily pattern of morphological changes of the L2 dendritic tree [8] but CRY also helps to maintain cycling of the Na^+^/K^+^-ATPase in the epithelial glial cells in the lamina.

It is uncertain whether there is regulation of lamina rhythms by the brain pacemaker because connections between the pacemaker neurons in the accessory medulla and the lamina have not been observed. We have found, however, that rhythms in axon plasticity of neurons in the lamina are circadian, have two peaks - morning and evening, and are synchronized with locomotor activity [7], [13], [57]. Our present results now show, that thin neurite extends from the aMe and arborizes in the distal lamina. In the aMe, the s-LN_v_s are regarded as the main pacemaker cells maintaining circadian rhythms [58]. The l-LN_v_s are involved in behavioral arousal and sleep [59], [60]. For these reasons, the LN_v_s are good candidates as oscillators controlling lamina rhythms. Moreover, all LN_v_s except the 5^th^ s-LN_v_, express PDF which may synchronize central oscillators with each other and with peripheral ones [34], [61]. In the housefly, large PDF-immunoreactive neurons, similar to *D. melanogaster*'s l-LN_v_s, have terminals in the lamina which show circadian structural changes [62]. Moreover, these neurons cyclically release PDF [63] that affects circadian plasticity in the lamina. In *D. melanogaster*, release of PDF from PDF-immunoreactive processes in the medulla, where these processes form a dense network of varicose processes, is also possible [64]. These processes, however, do not extend to the lamina. In the present study, PDF immunolabeling of the newly described *D. melanogaster*'s CRY-positive terminals in the lamina was negative. This does not exclude PDF action in the lamina, particularly when PDF receptors have been detected in non-neuronal cells between the lamina and the retina [34]. PDF may diffuse in the lamina after release from terminals in the distal medulla.

Ion transport peptide (ITP) and short neuropeptide F (sNPF) have been detected in the LN_v_s [37]. Among the five s-LN_v_s, ITP was found in the 5^th^ s-LN_v_, while sNPF was observed in four other s-LN_v_s which also express PDF. In the present study, we detected ITP-immunoreactive fibers, using the Schgr-ITP antisera, in the distal lamina, co-localized with *cry*-GAL4 driven GFP. The co-localization with ITP suggests that the projection into the lamina may originate from the 5^th^ s-LN_v_. Little is known about the function of the 5^th^ s-LN_v_. It has been suggested, that this neuron, together with LN_d_s and some DN_1_s, drive the evening peak of *D. melanogaster* bimodal activity [20], [21]. Our finding indicates a possible new function of the 5^th^ s-LN_v_ in regulating circadian structural rhythms in the lamina, since this neuron is immunoreactive to ITP. Like other peptides in the optic lobe [64], ITP seems to be released from varicose terminals in a paracrine way. We came to this conclusion because we did not detect synaptic contacts between ITP-immunoreactive processes and cells in the lamina. This peptide probably diffuses in the distal lamina and may facilitate chloride and/or other ion-dependent swelling and shrinking of the L1 and L2 axons. At least two ion pumps; the V-ATPase and Na^+^/K^+^-ATPase, show robust cyclical activity in the epithelial glial cells [55], [56]. The epithelial glial cells swell and shrink in anti-phase to the L1 and L2 interneurons [9]. Our preliminary results showed, that in a transgenic line carrying RNAi to block ITP expression, the pattern of rhythmic changes in the level of the α-subunit of the Na^+^/K^+^-ATPase in the lamina glial cells of *D. melanogaster* is different than the pattern in wild-type flies (Damulewicz M. and Pyza E., unpublished results). Thus, not only CRY but also ITP is important for maintaining rhythmic activity changes of the Na^+^/K^+^-ATPase.

The function of ITP in the nervous system is unknown. In the lamina ITP may play a similar regulatory role as in hindgut of insects, transporting ions and fluids across cell membranes [65], [66].

Since the L1 and L2 monopolar cells swell in the morning and in the evening, ITP released from the 5^th^ s-LN_v_ may drive the evening peak of this rhythm. This is thought to be so, because the 5^th^ s-LN_v_ and LN_d_ are regarded as the lateral neurons' evening oscillator. In turn, PDF may drive the morning peak because PDF is thought to control the morning peak of locomotor activity, in a LD 12∶12 regime [20], [21]. However, PDF's role in promoting locomotor activity in the evening has also been shown [67]. The role of ITP as a neurotransmitter of circadian information to the lamina and as a possible regulator of rhythmic swelling and shrinking of the L1 and L2 monopolar cells, requires more experimentation and will be the subject of the next study.

## Materials and Methods

### Animals

For the experiments, we used *D. melanogaster* Canton-S wild-type and transgenic lines: *cry*-GAL4 and UAS-S65T-GFP. To characterize cells with *cry*-active promoter, we used *cry-gal4(39)* [68], [48] (kindly donated by Dr. François Rouyer) expressing the yeast transcription factor gene *gal4*, under the control of the *cry* promoter crossed to the *UAS-S65T-gfp* line. In this line, the expression of GFP in cytoplasm is under the control of the UAS sequence. Virgin females of the *cry-gal4* strain were crossed to *UAS-S65T-gfp* males. In the first generation, progeny cells with the active *cry* promoter were labeled with GFP. Canton-S flies were used as the control. Flies were reared on a standard medium (cornmeal, agar, honey, yeast) in 25±1°C, in a LD 12∶12 light regime (12 h of light and 12 h of darkness). Males and females which were five days old were used for the experiments. Each experiment was repeated at least three times and the results were examined in 30 individuals at each time point.

### Immunohistochemistry

Flies were decapitated four times, to look for possible structural changes during the day of LD 12∶12 at: ZT1, ZT4, ZT13 and ZT16 (ZT0 – the beginning of the day, ZT12 – the beginning of the night). The flies were fixed in 4% paraformaldehyde in phosphate buffer saline (PBS; pH 7.4) for 4 h. Next, they were cryoprotected by incubating in 12.5% sucrose for 10 min and in 25% sucrose at 4°C overnight. Heads were embedded in Tissue Tek, frozen in liquid nitrogen, and cryostat 20 µm sections were cut. Alternatively, heads were fixed for 4 h, washed in PBS, and cut with vibratom on 100 µm sections. The sections were washed in PBS for 30 min. and 5 times in phosphate buffer (PB) with an addition of 0.2% TritonX100 (PBT). After that, sections were incubated in 5% normal goat serum (NGS) with an addition of 0.5% Bovine Serum Albumin (BSA) for 30 min first at room temperature, and then incubating the brain tissues with primary antibodies for 24 h (Table 1). Afterwards, sections were washed 6 times in PBT/BSA, and blocked in 5% NGS for 45 min. After that, secondary antibodies were applied overnight in 4°C (Table 2). Finally, sections were washed twice in BSA, 6 times in PBT, and twice in PBS. Then, cryosections or vibratom sections were mounted in Vectashield medium (Vector) and examined with a Zeiss Meta 510 Laser Scanning Microscope. Confocal images of 100 µm vibratom or 20 µm frozen sections were captured at 0.47 µm and 1 µm intervals, respectively, and viewed as Z-stacks. To measure differences at four ZTs in the fluorescence intensity of GFP in CRY-positive cells, we used the same parameters for brightness, contrast and other image settings. The fluorescence intensity of GFP in selected cells was measured using the already described methods [55]. For a particular cell, the mean level of fluorescence intensity was converted to the Mean Gray Value of that cell and quantified using ImageJ v. 1.4 software (NIH, Bethesda).

**Table 1:** The primary antibodies used in the study.

Antigens	Antisera	Dilution	Source
Green Fluorescent Protein	Rabbit polyclonal anti-GFP	1∶1,000	Novus BiologicalNo NB 600-308
Green Fluorescent Protein	Mouse monoclonal anti-GFP	1∶1,000	Novus BiologicalNo NB 600-597
PDF (Pigment Dispersing Factor)	PDFc7, mouse monoclonal	1∶1,000	Hybridoma
BRP (Bruchpilot)	Nc82 mouse monoclonal	1∶30	Hybridoma
REPO	8D12 mouse monoclonal	1∶300	Hybridoma
DVMAT (*Drosophila* Vesicular Monoamine Transporter)	Rat anti-DVMAT	1∶200	Provided by Dr. Bernhard T. Hovemann (Ruhr-Universität Bochum, Germany)
ITP (Ion Transport Peptide)	Rabbit anti-ITP	1∶1,000	Provided by Dr. Neil Audsley (The Food and Environment Research Agency, Sand Hutton, UK). The antibody was raised against a specific region of *Schistocerca gregaria* ITP (residues 60–67; DEEEKFNQ) so that it will not cross-react with ITP-L.
ITP-L	Rabbit anti-ITP-L	1∶1,000	Provided by Dr. Neil Audsley. The antibody was made to residues 65–79 (IQSWIKQIHGAEPGV) of *S. gregaria* ITP
sNPF (Short Neuropeptide F – sNPF-3 and -4)	Rabbit anti-RLRWamide	1∶1,000	Provided by Dr. Jan A. Veenstra (Université Bordeaux, France). The antibody was raised against the peptide RLRWamide.

**Table 2:** The secondary antibodies used in the study.

Antisera	Dilution	Source
Goat anti-rabbit conjugated with Alexa 488	1∶1,000	Molecular Probes
Goat anti-mouse conjugated with Alexa 514	1∶500	Invitrogen
Goat anti-rat conjugated with Cy3	1∶500	Jackson Immuno Research
Goat anti-mouse conjugated with Cy3	1∶500	Jackson Immuno Research
Goat anti-rabbit conjugated with Cy3	1∶300	Jackson Immuno Research
